# Definition of Eight Mulberry Species in the Genus *Morus* by Internal Transcribed Spacer-Based Phylogeny

**DOI:** 10.1371/journal.pone.0135411

**Published:** 2015-08-12

**Authors:** Qiwei Zeng, Hongyu Chen, Chao Zhang, Minjing Han, Tian Li, Xiwu Qi, Zhonghuai Xiang, Ningjia He

**Affiliations:** State Key Laboratory of Silkworm Genome Biology, Institute of Sericulture and Systems Biology, Southwest University, 216 Tiansheng Road, Beibei District, Chongqing, 400716, China; Zhejiang University, CHINA

## Abstract

Mulberry, belonging to the order Rosales, family Moraceae, and genus *Morus*, has received attention because of both its economic and medicinal value, as well as for its important ecological function. The genus *Morus* has a worldwide distribution, however, its taxonomy remains complex and disputed. Many studies have attempted to classify *Morus* species, resulting in varied numbers of designated *Morus* spp. To address this issue, we used information from internal transcribed spacer (ITS) genetic sequences to study the taxonomy of all the members of generally accepted genus *Morus*. We found that intraspecific 5.8S rRNA sequences were identical but that interspecific 5.8S sequences were diverse. *M*. *alba* and *M*. *notabilis* showed the shortest (215 bp) and the longest (233 bp) ITS1 sequence length, respectively. With the completion of the mulberry genome, we could identify single nucleotide polymorphisms within the ITS locus in the *M*. *notabilis* genome. From reconstruction of a phylogenetic tree based on the complete ITS data, we propose that the *Morus* genus should be classified into eight species, including *M*. *alba*, *M*. *nigra*, *M*. *notabilis*, *M*. *serrata*, *M*. *celtidifolia*, *M*. *insignis*, *M*. *rubra*, and *M*. *mesozygia*. Furthermore, the classification of the ITS sequences of known interspecific hybrid clones into both paternal and maternal clades indicated that ITS variation was sufficient to distinguish interspecific hybrids in the genus *Morus*.

## Introduction

Mulberry (*Morus* spp.), belonging to the order Rosales, family Moraceae, and genus *Morus* [1], is distributed in a wide range of areas worldwide [2]. It is an important economic woody plant with a significant impact on human society due to its economic value in sericulture as well as for its nutritional benefits and medicinal values [3]. This plant also plays significant ecological roles in the prevention and control of sand erosion, and in stony desertification and saline-alkali land treatments [4]. However, the taxonomy of *Morus* has been disputed [5] because of its wide geographical distribution, morphological plasticity [6], hybridization among species [7], long history of domestication, and introduction and naturalization of species [8]. Different studies have recognized a variable number of *Morus* species, leading to an uncertain taxonomy. In 1753, Linnaeus established the first taxonomy of *Morus* [9]. The first comprehensive *Morus* taxonomy was created by Bureau [10] based on features of its leaves and pistillate catkins, and recognized five species, 19 varieties, and 13 sub-varieties. Since that time, the taxonomy has undergone many further modifications based on morphological and phenological characteristics [5]. In 1917, Schneider [11] described a new species, *Morus notabilis* C. K. Schneid in China. Subsequently, Koidzumi [12], Leroy [13], and Hotta [14] classified the genus *Morus* into 24 species and 1 subspecies, 19 species, and 35 species, respectively. More recently, Zhou and Gilbert [15] described 16 species in *Morus*, 12 of which were found in China. Because of this variable taxonomy, 260 validated published names in *Morus* have been deposited in the International Plant Names Index (http://www.ipni.org/), with many of them being synonymous. Therefore, although as many as 68 [16,17] or 150 [18,19] species have been reported in *Morus*, only 10–16 are generally cited and accepted [20–24].

Molecular markers that are heritable, fast, and easy to measure and evaluate have been used to classify *Morus* species in order to improve the taxonomy of *Morus* based on phenotypic characteristics. Using amplified fragment length polymorphism (AFLP) markers, Sharma [25] and Kafkas [26] concluded that the results from both marker and conventional methods were consistent to a large extent and that *M*. *nigra* and *M*. *rubra* were molecularly distinct from *M*. *alba*. Rao [27] grouped domesticated species (*M*. *alba* and *M*. *indica*) into one cluster by building a phylogenetic tree of 80 wild mulberries from four species using four isozymes (polyphenol oxidase [PPO], peroxidase [PoX], esterase [EST], and diaphorase [DIA]). Zhao [28–32] placed Chinese domesticated species, including *M*. *alba*, *M*. *multicaulis*, *M*. *bombycis*, *M*. *australis*, *M*. *atropurpurea*, and *M*. *rotundiloba*, into one cluster by creating phylogenetic trees using ITS sequences, microsatellite loci, and sequence related amplified polymorphism (SRAP), inter simple sequence repeat (ISSR), and SSR markers. Recently, the genus *Morus* was classified into 13 species using a combination of ITS and *trnL-trnF* intergenic spacer markers, these included eight Asian species (*M*. *alba*, *M*. *australis*, *M*. *cathayana*, *M*. *macroura*, *M*. *mongolica*, *M*. *nigra*, *M*. *notabilis*, and *M*. *serrata*), four New World species (*M*. *celtidifolia*, *M*. *insignis*, *M*. *microphylla*, and *M*. *rubra*), and one African species (*M*. *mesozygia*) [24]. However, the *M*. *notabilis* sample they collected was not a true *M*. *notabilis* sample because the ITS sequence (GenBank accession number HM747175) is actually identical to that of *M*. *alba*. The fact that these studies did not include *M*. *notabilis*, as reported by Schneider [11], limits our full understanding of *Morus* taxonomy.

In plants, the ITS locus has been widely used in phylogenetic inference [33] and has shown the highest discriminatory power for species [34]. Furthermore, ITS has been recently proposed for use as the primary DNA barcode marker in fungi [35] and its use has enabled the estimation of highly accurate phylogenies of closely related organisms in yeast [36]. In this study, which includes data from *M*. *notabilis*, we characterized the ITS locus in *Morus* spp. including length and SNP. Using this ITS data, we redefined the taxonomy of *Morus* into eight species (*M*. *alba*, *M*. *nigra*, *M*. *notabilis*, *M*. *serrata*, *M*. *celtidifolia*, *M*. *insignis*, *M*. *rubra*, and *M*. *mesozygia*).

## Materials and Methods

### Plant materials

All the Plant materials used in this study were listed in the Table 1. *M*. *notabilis* located at 29°45.278' north latitude, 102°53.878' east longitude, a wild mulberry species with seven pairs of chromosomes, was collected by the author (*Ningjia He*) from a pristine forest in Ya’an, Sichuan province, Southwest China. *M*. *yunnanensis* was kindly provided by associate-researcher Yining Chu from the Institute of Sericulture and Apiculture, Yunnan Academy of Agricultural Sciences, Mengzi, Yunnan. *M*. *nigra* located at 36°59.606' north latitude, 81°14.651' east longitude, was collected by the author (*Qiwei Zeng*) from Yutian County, Xinjiang Uygur Autonomous Region, China. The collection locations of *M*. *notabilis* and *M*. *nigra* in this study are not protected lands. Other samples were from the Mulberry Germplasm Nursery in Southwest University, China. No special permits were required for the collection of the additional *Morus* spp. for this study as these species of *Morus*, which is distributed worldwide, are not considered to be endangered and are not listed as protected species, and the samples were obtained from public lands without restrictive ordinance (*M*. *alba*, *M*. *mongolica*, *M*. *macroura*, *M*. *cathayana*, *M*. *australis*, *M*. *wittiorum*, *M*. *bombycis*, *M*. *indica*, *M*. *multicaulis*, and *M*. *atropurpurea*).

**Table 1:** Plant materials used in this study.

GenBank accession numbers	Species	Origins	Chromosome number (Ploidy)	Brief morphological characters
AB564722	***C*. *sativa***	Asia	20(2X)	Female flowers green and sessile, the broader leaves are made up of two or more discrete leaflet, leaflet usually lanceolate to linear, margin deeply serrated.
AM041997	***M*. *indica***	Asia	28(4X)	Female flowers with style long, leaf margin sharply serrate often deeply lobed, scaberulous, female spikes short ovoid.
AM041999	***M*. *lhou***	Asia	28(4X)	Female flowers with absent style, leaf blade thick, wrinkled with sparsely pubescent, syncarp greenish white to purple when mature.
AM042004	***M*. *australis***	Asia	28(4X)	Female flowers with long style, leaf lobes rounded to linear, base cuneate to cordate, margin serrate.
AM042005	***M*. *rotundiloba***	Asia	28(4X)	Female flowers with long style, stigma lancecolate, minutely papillose, leaf trilobate and apex of the lateral lobes is rotundate.
AM042006	***M*. *bombycis***	Asia	42(6X)	Female flowers with style long and conspicuous, leaf blade marginal teeth and stigmas without a nipple-like protuberance.
AY345145	***M*. *atropurpurea***	Asia	42(6X)	Female flowers with absent style, bark gray, leaf blade thin and flat, syncarp blackish purple when mature, ovoid to ellipsoid.
AY345154	***M*. *wittiorum***	Asia	28(4X)	Female flowers with very short style, bark grayish white and smooth, leaf blade oblong to broadly elliptic, syncarp 10–16 cm.
EU091563	***F*. *adhatodifolia***	Asia	26(2X)	Female flowers ovary white or reddish at base, leaf blade margin entire with slightly scabrous, wax glands absent or in axils of main basal veins.
F784877	***M*. *notabilis***	Asia	14(2X) *	Female flowers with long style, unisexual flower, leaf not lobed, bark grayish brown, syncarp white when mature, 3.5–4 cm.
FJ980402	***M*. *alba***	Asia	28(4X)	Female flowers with absent style, leaf adaxially bright green and glabrous, abaxially pubescent along the veins, bark gray.
HM623778	***B*. *papyrifera***	Asia	26(2X)	Female flowers with style, bark dark gray, leaf blade ovate to elliptic-ovate, abaxially densely pubescent, base cordate and asymmetric, margin coarsely serrate.
HM747167	***M*. *cathayana***	Asia	28(4X)	Female flowers with short style, leaf sometimes lobed, thick papery and adaxially scabrous, syncarp white, red, or dark purple when mature, 2–3 cm.
HM747168	***M*. *celtidifolia***	America	28(4X)	Female flower with short style, leaf abaxially harshly pubescent to scabrous and adaxially slightly scabrous, base usually unequal to cordate, bark gray.
HM747169	***M*. *insignis***	America	84(12X)	Female flower with short style, leaf lanceolate to elliptic, margin minutely serrate to subentire, syncarp 5–16 cm or longer.
HM747170	***M*. *macroura***	Asia	28(4X)	Female flowers with absent style, margin minutely and densely serrate, abaxially pale green and adaxially dark green, syncarp yellowish white when mature, 6–12 cm.
HM747171	***M*. *mesozygia***	Africa	28(4X)	Female flowers with very short style, leaves ovate or elliptic, glossy dark green above, distinctly 3-veined from the base, margin serrate, unisexual flower, ripe syncarps pale green.
HM747173	***M*. *mongolica***	Asia	28(4X)	Female flowers with long style, leaf blade elliptic-ovate and base cordate stigmas with a nipple-like protuberance.
HM747176	***M*. *serrata***	Asia	84(12X)	Female flowers with absent style, leaf blade broadly ovate, unlobed and base cordate, margin toothed, and syncarp red when mature.
HQ144170	***M*. *alba* x *M*. *rubra***	America	28(4X)	The intermediate phenotype between *M*. *alb*a and *M*. *rubra*, easily misidentified by current morphological criteria.
HQ144171	***M*. *alba* x *M*. *rubra***	America	28(4X)	The intermediate phenotype between *M*. *alb*a and *M*. *rubra*, easily misidentified by current morphological criteria.
HQ144175	***M*. *alba* x *M*. *rubra***	America	28(4X)	The intermediate phenotype between *M*. *alb*a and *M*. *rubra*, easily misidentified by current morphological criteria.
HQ144180	***M*. *rubra***	America	28(4X)	Female flower with absent or indistinct style, leaf surface adaxially rough and dull green, abaxially densely pubescent, sometimes lobed, leaf margin regularly serrated, grayish bark and syncarp deep purple to red.
HQ144187	***M*. *alba* x *M*. *rubra***	America	28(4X)	The intermediate phenotype between *M*. *alb*a and *M*. *rubra*, easily misidentified by current morphological criteria.
KF784875	***M*. *nigra***	Asia	308(44X)*	Female flowers with inconspicuous style, bark dark brown, leaf blade broadly ovate, thick and base cordate, syncarp blackish purple when mature, elliptic, 1.5–2.5 cm in diam.
KF784879	***M*. *mongolica***	Asia	84(12X) *	Female flowers with long style, leaf blade elliptic-ovate and base cordate stigmas with a nipple-like protuberance.
KF784881	***M*. *alba***	Asia	28(4X)	Female flowers with absent style, leaf adaxially bright green and glabrous, abaxially pubescent along the veins, bark gray.
KF784882	***M*. *cathayana***	Asia	28(4X)	Female flowers with short style, leaf sometimes lobed, thick papery and adaxially scabrous, syncarp white, red, or dark purple when mature, 2–3 cm.
KF784883	***M*. *multicaulis***	Asia	28(4X) *	Female flowers with absent style, leaf blade thick, wrinkled with sparsely pubescent, syncarp greenish white to purple when mature.
KF784884	***M*. *alba***	Asia	28(4X)	Female flowers with absent style, leaf adaxially bright green and glabrous, abaxially pubescent along the veins, bark gray.
KF784885	***M*. *alba***	Asia	28(4X)	Female flowers with absent style, leaf adaxially bright green and glabrous, abaxially pubescent along the veins, bark gray.
KF784886	***M*. *wittiorum***	Asia	28(4X)	Female flowers with very short style, bark grayish white and smooth, leaf blade oblong to broadly elliptic, syncarp 10–16 cm.
KF784887	***M*. *alba***	Asia	28(4X)	Female flowers with absent style, leaf adaxially bright green and glabrous, abaxially pubescent along the veins, bark gray.
KF784888	***M*. *atropurperea***	Asia	42(6X)	Female flowers with absent style, bark gray, leaf blade thin and flat, syncarp blackish purple when mature, ovoid to ellipsoid.
KF784889	***M*. *australis***	Asia	28(4X)	Female flowers with long style, leaf lobes rounded to linear, base cuneate to cordate, margin serrate.
KF784890	***M*. *indica***	Asia	28(4X) *	Female flowers with long style, leaf margin sharply serrate often deeply lobed, scaberulous, female spikes short ovoid.
KF784891	***M*. *alba***	Asia	28(4X)	Female flowers with absent style, leaf adaxially bright green and glabrous, abaxially pubescent along the veins, bark gray.
KF784892	***M*. *atropurpurea***	Asia	28(4X)	Female flowers with absent style, bark gray, leaf blade thin and flat, syncarp blackish purple when mature, ovoid to ellipsoid.
KF784893	***M*. *atropurpurea***	Asia	28(4X)	Female flowers with absent style, bark gray, leaf blade thin and flat, syncarp blackish purple when mature, ovoid to ellipsoid.
KF784894	***M*. *atropurpurea***	Asia	28(4X) *	Female flowers with absent style, bark gray, leaf blade thin and flat, syncarp blackish purple when mature, ovoid to ellipsoid.
KF784895	***M*. *alba***	Asia	28(4X)	Female flowers with absent style, leaf adaxially bright green and glabrous, abaxially pubescent along the veins, bark gray.
KF784896	***M*. *alba***	Asia	28(4X)	Female flowers with absent style, leaf adaxially bright green and glabrous, abaxially pubescent along the veins, bark gray.
KF784897	***M*. *atropurpurea***	Asia	28(4X) *	Female flowers with absent style, bark gray, leaf blade thin and flat, syncarp white when mature, ovoid to ellipsoid.
KF850474	***M*. *yunnanensis***	Asia	14(2X) *	Female flowers with long style, leaf not lobed, bark grayish brown, syncarp white when mature, 3.5–4 cm.

Note: *M*., *Morus*; *B*., *Broussonetia*; *F*., *Ficus*; *C*., *Cannabis*; The ploidy of *Morus* is based on the recent studies that the cardinal chromosome number of *Morus* is 7 (X = 7), asterisks represent the chromosome number identified by our lab; “Brief morphological characters of *Morus”* are mainly adapted from Flora of China, Flora of North America, Nepal’s paper (Nepal and Ferguson, 2012). The plant materials were listed alphabetically according to GenBank accession numbers.

### DNA isolation, amplification, and sequencing

Total DNA was extracted from fresh leaves using a DNeasy plant mini kit (Qiagen Corp., Valencia, California, USA). The target sequence regions (the partial 18S rRNA gene, ITS1, 5.8S rRNA gene, ITS2, and the partial 28S rRNA gene) were amplified with mulberry-specific forward (5′-GTA ACA AGG TTT CCG TAG GTG-3′) and reverse (5′-TAA ACT CAG CGG GTA GCC-3′) primers, which were designed from the highly conserved regions flanking the 3′ end of the 18S rRNA gene and the 5′ end of the 28S rRNA gene, respectively, based on available *Morus* rRNA gene sequences in GenBank (National Center for Biotechnology Information (NCBI), http://www.ncbi.nlm.nih.gov/). Polymerase chain reaction (PCR) amplification was conducted in a reaction mixture of 20 μL containing 20 ng genomic DNA, 2 mM PCR buffer, 0.2 mM each primer, 0.2 mM each dNTP, and 1 unit Taq polymerase with 1.25 mM or 2.5 mM MgCl_2_ (Takara Bio-technology (Dalian) Co. Ltd., Dalian, China). The PCR amplification protocol was as follows: 94°C for 5 min, followed by 30 cycles of 94°C for 30 s, 58°C for 30 s, and 72°C for 1 min, followed by a final 10 min extension at 72°C. The PCR products were separated on a 1% agarose gel. The target fragment was gel-purified using the Takara Agarose Gel DNA Purification Kit (Takara Bio-technology (Dalian) Co. Ltd., Dalian, China), cloned into the vector pUCm-T (Sangon, Shanghai, China) by AT cloning according to manufacturer’s instructions, and individual clones were sequenced by BGI-Shenzhen (Shenzhen, China).

### Data analyses

We downloaded an additional 187 *Morus* ITS sequences from NCBI (http://www.ncbi.nlm.nih.gov/), and determined the sequence boundaries of ITS1, 5.8S, and ITS2 within all *Morus* sequences by comparison to known *Arabidopsis thaliana* ITS sequences (X52320). All data containing the full ITS1, 5.8S rRNA and ITS2 sequences were used for analysis. The sequence length and GC contents of all samples were analyzed by PerlScript.

### Detection of SNPs in *M*. *notabilis*

In order to detect *M*. *notabilis* ITS SNPs, the improved Short Oligonucleotide Alignment Program (SOAP2) [37] was used to map all of the *M*. *notabilis* genome reads from 12 libraries [38] onto the reference sequence, a 3709-bp fragment located at scaffold2247 (GenBank accession number KF784877). SNPs were detected using SoapSNP essentially as described [37], but with the following modifications: SoapSNP calls were discarded if the quality score of the consensus genotype was less than 20, if the sequencing depth of the site was less than 5, or if the distance of a neighbor candidate SNP was less than 5.

### Phylogenetic analysis and estimation of divergence times

Phylogenetic analysis was performed using a Bayesian method with MrBayes V3.1.2 software [39]. The phylogenetic tree of the ITS locus (ITS1-5.8S-ITS2) was rooted by the corresponding homologous sequences in *Broussonetia papyrifera* and *Ficus adhatodifolia*, which are sister genera of *Morus*, and in *Cannabis sativa*, which is another member of the Rosales order in the Cannabaceae. The phylogenetic tree was reconstructed and the estimations of the divergence times were determined according to the method described by Sun et al [40]. The divergence time of *M*. *notabilis* and *C*. *sativa* [38] was used to calibrate the estimation.

## Results

### Sequence characteristics of the ITS region

From our analysis, an additional 187 ITS sequences of *Morus* that included generally accepted *Morus* species were deposited in NCBI, including 56 sequences that contained the complete ITS region (ITS1-5.85S-ITS2), 60 that contained the partial 18S region (3′ end, “AGG ATC ATT G”), 156 covering the complete 5.8S and ITS2 region, and 57 sequences that contained the partial 28S region (5′ end, “G(T/C)G ACC CCA G”). After adding the newly sequenced ITS regions, we characterized the structures of the ITS nucleotide sequences (Table 2) along with their sequence lengths and GC contents (Table 3). The first 10 nucleotides of the ITS1 region were highly conserved in *Morus*, and a transversion (G/T) and a single nucleotide deletion (T) were found in the last 10 nucleotides (Table 2). The first transition of the ITS region (A/G, where G was only identified in *M*. *rubra*, *M*. *serrata*, and *M*. *insignis*) was detected in the 13th nucleotide. The first 10 nucleotides in the 5.8S region were also highly conserved, with the exception of a single-nucleotide substitution (A/T) at the first position of *M*. *notabilis* [38] (GenBank accession number KF784877) and *M*. *yunnanensis* (GenBank accession number KF850474) and a transition (T/C) at the fifth position of *M*. *mesozygia* (GenBank accession number HM747171). The last 10 nucleotides of 5.8S exhibited a transition (C/T) and a transversion (A/C) at 4th and 9th positions, respectively. In total, there were 8 variable positions in the 5.8S region in *Morus*. The 5.8S subunits of 121 *Morus* spp. including *M*. *alba*, *M*. *mongolica*, *M*. *macroura*, *M*. *cathayana*, *M*. *australis*, *M*. *wittiorum*, *M*. *bombycis*, *M*. *indica*, *M*. *multicaulis* (*M*. *lhou*), and *M*. *atropurpurea* were uniform in size (163 bp) with a GC content of 52.147%, except for *M*. *australis* (GenBank accession number AM042004), which had a GC content of 51.533%. In the genus *Morus*, *M*. *alba* and *M*. *notabilis* contained the shortest (215 bp) and longest (233 bp) respective ITS1 sequence lengths, and *M*. *mesozygia* and *M*. *notabilis* (or *M*. *celtidifolia*) contained the shortest (217 bp) and longest (235 bp) ITS2 sequence lengths, respectively (Table 3). In contrast to other *Morus* species, a 13 bp deletion of the ITS1 region in *M*. *alba* was located at the 55th nucleotide position. The comparison of the 5.8S region sequences of *M*. *alba* (121 sequences), *M*. *rubra* (9 sequences), *M*. *notabilis* (6 sequences), and *M*. *celtidifolia* (4 sequences) found that the intraspecific 5.8S regions of these species were completely identical. In addition, we found that the ITS1 sequences of interspecific hybrids between *M*. *alba* and *M*. *rubra* consisted of 215 bp (GenBank accession numbers HQ144171 and HQ144170) and 229 bp (GenBank accession numbers HQ144175 and HQ144187) sequences, respectively. However, the ITS2 lengths of these hybrids were unique (Table 3).

**Table 2:** Nucleotide sequences at the beginning and end of the *Morus* ITS region. Sequence length is 10 bp. ITS, internal transcribed spacers.

Region	Sequence starts with (5′–3′):	Sequence ends at (5′–3′):
**ITS1**	TCG AAA CCT G	(G/C)TT (T)AA GTC T
**5.8S**	(A/T)AA A(T/C)G ACT C	GGG (C/T)GT CA(A/C) A
**ITS2**	CAC CG(T/A) TGC C	TGC (C/T)T(C/T) (T/C) GA (T/C)

**Table 3:** Sequence length (bp) and percentage GC content (100%) of the ITS region in the genus *Morus*.

Taxon	ITS1	GC	5.8S	GC	ITS2	GC	Length
***M*. *alba***	215	59.5	163	52.1	233	63.5	611
***M*. *celtidifolia***	228	59.2	163	52.1	235	62.5	626
***M*. *rubra***	229	60.4	163	50.6	233	61.8	625
***M*. *notabilis***	233	59.2	163	52.2	235	63.4	631
***M*. *insignis***	229	64.2	163	52.8	232	65.3	624
***M*. *mesozygia***	231	60.2	163	53.4	217	63.1	607
***M*. *serrata***	229	60.3	163	50.9	233	61.8	625
***M*. *nigra***	228	59.4	163	52.1	233	61.3	624
***M*. *alba* x *M*. *rubra***	229 (215)	60.7 (59.5)	163	50.8 (52.1)	234 (232)	61.9 (62.7)	626 (610)

### Detection of SNPs in *M*. *notabilis* and ITS genetic heterogeneity in *Morus*

To detect SNP sites in the ITS region in the draft genome of *M*. *notabilis*, we employed the algorithm SOAP2 to map all of the genome reads, identifying six SNPs in the ITS region of *M*. *notabilis* (Table 4). In the *M*. *alba* lineage, only *M*. *australis* (GenBank accession number AM042004) showed variation in the 5.8S region. Comparison of all *M*. *australis* 5.8S region data deposited in GenBank indicated that ten sequences from different specimens were identical. In the full collection of ITS1 and ITS2 sequences of *M*. *alba*, we identified one SNP (A/G) existing at the 82nd site of ITS1 (4 out of 41 sequences) and one SNP (T/G) existing at the 219th site of ITS2 (21 out of 107 sequences). In *M*. *rubra*, 4 and 1 variable loci existed in the ITS1 and ITS2 regions of 9 sequences, respectively. Among these 5 variable loci, 4 occurred in two sequences from different samples, which suggested the possibility of 4 SNPs in the ITS region of *M*. *rubra*. In *M*. *celtidifolia*, we identified 3 variable loci in the ITS1 and ITS2 regions out of five ITS sequences.

**Table 4:** Predicted SNP sites in the ITS region of *M*. *notabilis*.

	Site*	SNP
**ITS1**	1973	C/T
	2025	G/A
**5.8S**	2120	G/A
**ITS2**	2220	A/C
	2309	G/A
	2330	T/C

*Sites are numbered from the beginning of the 18S rRNA sequence. SNP, single nucleotide polymorphism.

### Phylogenetic analysis and estimation of divergence time

**Fig 1:**
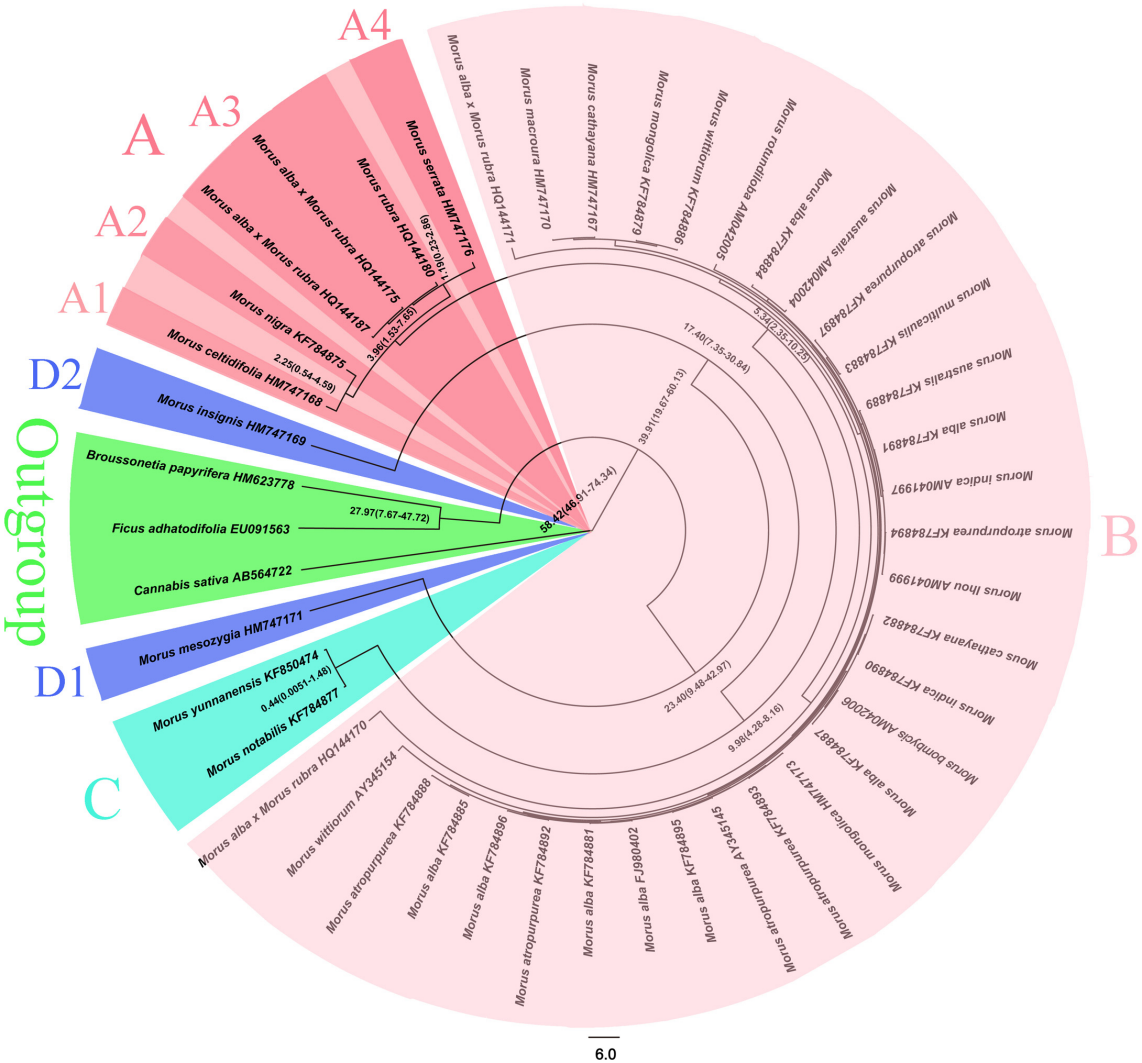
Phylogenetic tree and chronology of divergence times based on ITS as estimated using Bayesian methods implemented in BEAST. Assignments into clades A (light red; A1, A2, A3 and A4 represent *M*. *celtidifolia*, *M*. *nigra*, *M*. *rubra* and *M*. *serrata*, respectively), B (light pink; B represents *M*. *alba*), C (light sky blue; C represents *M*. *notabilis*), D (light blue, D1 and D2 represent *M*. *mesozygia* and *M*. *insignis*, respectively) and an outgroup (light green) are shown. Values represent the divergence times with the 95% highest posterior density intervals shown in parentheses.

Bayesian inference of phylogeny based on ITS sequences separated *Morus* species into four large clades, as shown in Fig 1. The A clade was a polyphyletic group comprised of two Asian species (*M*. *nigra* and *M*. *serrata*) and two American species (*M*. *rubra* and *M*. *celtidifolia*). The A clade also included two clones (GenBank accession numbers HQ144175 and HQ144187) that were a red mulberry parent of a hybrid (*M*. *rubra* x *M*. *alba*). The calculated estimated divergence time between *M*. *celtidifolia* and *M*. *nigra* was 2.25 Mya. The B clade, which was the largest clade, was a monophyletic taxa comprised of only Chinese white mulberry (*M*. *alba*). The B clade also included two clones (GenBank accession numbers HQ144170 and HQ144171, amplified from the same hybrid mentioned above), which were a white mulberry parent of the hybrid. The divergence time between the A and B clades was estimated to be 5.34 Mya. The C clade consisted of *M*. *notabilis* and *M*. *yunnanensis*, with a 0.44 Mya estimated divergence time. Similar to *M*. *notabilis*, *M*. *yunnanensis*, which used to be recognized as variety of *M*. *mongolica*, was also found to contain 14 chromosomes. Finally, the D clade consisted of two species including *M*. *insignis* and *M*. *mesozygia*. The estimate of the divergence time between the *Morus* genus and the *Ficus* and *Broussonetia* genera was 39.91 Mya.

## Discussion

Mulberry is not only an important economic woody tree [24] but also plays significant roles in the recovery and restoration of damaged ecosystems [4], and was planted by famers as early as 5000 years ago [38]. Although the genus *Morus* is widely cultivated in the Eurasian, African, and American continents, its taxonomy is complex and has been disputed. Since Linnaeus [9], researchers have used morphological structures of the leaf, winter bud, bark, pistil, syncarp, and leaf idioblasts to infer evolutionary relationships among *Morus* species [10–14].

DNA-based markers have been developed to better classify *Morus* species [24–32,41]. Currently, ITS data have the highest discriminatory power for plant species determination [34] and this marker is widely used for taxonomic and phylogenetic analyses [42,43]. Although ITS data have previously been used to classify *Morus* species [24,28,44–46], one recognized species, *M*. *notabilis* [11], was not included in these studies. In this study, we supplemented the 24 ITS sequences (GenBank accession numbers including KF784875-KF784897, KF850474) of *Morus* by including data for *M*. *notabilis* and *M*. *yunnanensis* (14 chromosomes), and *M*. *nigra* (308 chromosomes) collected using the method of cloning and sequencing.

Previously reported lengths of the *Morus* 5.8S gene were 152, 159, or 100 bp [28,45,47]. Here, we found the 5.8S length to be 163 bp, which was consistent with that of other species such as *Malus*, *Triticum*, and *Canella* [48–54]. We also found that the samples with a 215 bp ITS1 sequence carried identical 5.8S sequences, these included *M*. *alba*, *M*. *mongolica*, *M*. *cathayana*, *M*. *australis*, *M*. *macroura*, *M*. *wittiorum*, *M*. *bombycis*, *M*. *indica*, *M*. *trilobata*, and *M*. *multicaulis*. To explore this phenomenon, we sorted the species names of these samples and found that all of them are synonyms or subspecies/varieties of *M*. *alba* [10,15,19,47]. Currently, there are 10–16 generally accepted *Morus* species, including *M*. *alba*, *M*. *australis*, *M*. *cathayana*, *M*. *macroura*, *M*. *mongolica*, *M*. *nigra*, *M*. *notabilis*, *M*. *serrata*, *M*. *celtidifolia*, *M*. *insignis*, *M*. *microphylla*, *M*. *rubra*, *M*. *mesozygia*, *M*. *bombycis*, *M*. *wittiorum*, and *M*. *trilobata* [15,24]. *M*. *mongolica*, *M*. *cathayana*, *M*. *australis*, *M*. *wittiorum*, and *M*. *macroura* used to be widely accepted as distinct *Morus* species, however, in this study, we found that they should be attributed to *M*. *alba*. Furthermore, we found that the 5.8S sequences of *M*. *notabilis* (6 clones, originating from different samples), *M*. *rubra* (9 clones from different samples), and *M*. *celtidifolia* (4 clones from different samples) were also identical. *M*. *insignis* (GenBank accession number HM747169), *M*. *mesozygia* (GenBank accession number HM747171), and *M*. *serrata* (GenBank accession number HM747176) had only one ITS sequence each deposited in NCBI, so it was necessary to obtain additional data to test the identification of their 5.8S sequences. Based on characterization of the 5.8S sequences from *M*. *alba*, *M*. *rubra*, *M*. *notabilis*, and *M*. *celtidifolia*, we found that the intraspecific 5.8S sequence was invariable in these species, whereas the interspecific 5.8S sequences were variable in *Morus* species exhibiting no evidence of hybridization. Therefore, the inclusion of the 5.8S region as part of the ITS sequence is critically important for taxonomic purposes [34].

In the present study, we found that *M*. *serrata* (GenBank accession number HM747176), referred to as *M*. *serrata Roxb* and also known as the Himalayan Mulberry [55], was distinct from *M*. *serrata* Wall., a synonym of *M*. *alba*. *M*. *yunnanensis* was previously regarded as a variant of *M*. *mongolica*, but *M*. *yunnanensis* Koidz. S. S. Chang was a synonym for *M*. *notabilis*, which was supported by our results. In addition, *M*. *microphylla* was used as a synonym for *M*. *celtidifolia* [56]. Therefore, with no regard for synonyms, subspecies, or varieties, the generally accepted *Morus* species identified in our study included four species native to Asia (*M*. *alba*, *M*. *serrata*, *M*. *nigra*, and *M*. *notabilis*), three New World species (*M*. *celtidifolia*, *M*. *insignis*, and *M*. *rubra*), and one species from Africa (*M*. *mesozygia*).

The use of ITS as a species barcode has been criticized due to its limitations, which include intraspecific variation [42] and heterogeneous copies [43,57]. However this heterogeneity did not affect the molecular identification of species [57]. The heterogeneity of ITS could be detected by both direct sequencing and sequencing after cloning [42]. In this study, we analyzed the heterogeneity of four *Morus* species. The results showed that there were SNPs in the ITS regions of *M*. *notabilis*, *M*. *alba*, *M*. *rubra*, and *M*. *celtidifolia*. Among these, the proportion of SNPs in the ITS region of *M*. *notabilis* was similar to that in the entire *M*. *notabilis* genome [38]. For *Morus* species without genome samples or repetitive ITS data in GenBank, ITS heterogeneity cannot be studied. Although intraspecific ITS SNPs were detected in *Morus* spp., the lengths of their intraspecific ITS regions and the positions of the *Morus* spp. with intraspecific ITS SNPs in the tree were invariable.

In this study, we demonstrated that interspecific hybrids of *Morus* species could be identified by ITS sequences. For example, two *M*. *alba* (GenBank accession numbers HQ144171 and HQ144170) and two *M*. *rubra* (GenBank accession numbers HQ144175 and HQ144187) clones were detected in a hybrid sample. Another example was an ITS sequence of *M*. *rubra* (FJ605516) whose sequence was identical to sequences of a hybrid (HQ144170) in the ITS1 and 5.8S regions, except for five separate locus differences in the ITS2 region. This suggested that the sample was possibly a hybrid (*M*. *rubra* x *M*. *alba*) because interspecific hybrids are quite commonly misidentified by morphological criteria [58].

We reconstructed the phylogenetic tree of the genus *Morus* based on ITS sequences. According to the phylogenetic analysis, the largest clade, B, was defined by a 215 bp ITS1 sequence and was a monophyletic group (*M*. *alba*), which agreed with the results obtained from sorting *Morus* species names. The C clade included *M*. *notabilis* from Ya’an county and *M*. *yunnanensis* from Dawei Mountain in China, each of which contained 14 chromosomes and an ITS1 sequence length of 233 bp. The distribution of parents of interspecific hybrids into separate clades (GenBank accession numbers HQ144175 and HQ144187, HQ144171 and HQ144170) indicated that ITS could be used to investigate *Morus* interspecific hybrids. The results of the phylogenetic analysis also indicated that there were only eight species in the genus *Morus*. Furthermore, the calibrated point of 63.5 (46.9–76.6) Mya for estimating the divergence time of the genus *Morus* was adopted from the mulberry genome [38]. This calibrated point is consistent with the divergence time of Moraceae (55 Mya) estimated by nuclear and chloroplast DNA analysis combined with data from multiple fossils [59], which confirmed that the estimated divergence time of the *Morus* genus was reliable.

Therefore, from the results obtained in this study after including new data for ITS sequences and reconstructing the phylogenetic tree of the genus *Morus*, we suggest that the genus *Morus* can be classified into eight species and that ITS might to be used to discover hybridization in this genus.

## Supporting Information

S1 Dataset (DOCX)
