# Supplementary material for: Definition of Eight Mulberry Species in the Genus Morus by Internal Transcribed Spacer-Based Phylogeny
Source: PLoS One. 2015 Aug 12;10(8):e0135411. doi: 10.1371/journal.pone.0135411 (PMC4534381; doi:10.1371/journal.pone.0135411)
Supplement: S1 Dataset — (DOCX) [file pone.0135411.s001.docx]

The following 187 GenBank accession numbers were used in this study including:

HQ144187.1, HQ144175.1, HQ144171.1, AY345145.1, KF850474.1, KF784897.1, KF784896.1, KF784895.1, KF784894.1, KF784893.1, KF784892.1, KF784891.1, KF784890.1, KF784889.1, KF784888.1, KF784887.1, KF784886.1, KF784885.1, KF784884.1, KF784883.1, KF784882.1, KF784881.1, KF784880.1, KF784879.1, KF784878.1, KF784877.1, KF784876.1, KF784875.1, HM747172.1, HQ144186.1, HQ144185.1, HQ144184.1, HQ144170.1, AY345153.1, AY345149.1, AY345147.1, KC539580.1, HM747176.1, HM747175.1, HM747174.1, HM747173.1, HM747171.1, HM747170.1, HM747169.1, HM747168.1, HM747167.1, HM747166.1, HM747165.1, HM747164.1, KF672603.1, HQ144183.1, HQ144182.1, HQ144181.1, HQ144180.1, HQ144179.1, HQ144178.1, HQ144177.1, HQ144176.1, HQ144174.1, HQ144173.1, HQ144172.1, AY345158.1, AY345157.1, AY345155.1, AY345154.1, AY345152.1, AY345151.1, AY345150.1, AY345148.1, AY345146.1, FJ599759.1, KJ605416.1, FJ605516.1, FJ605515.1, KF672604.1, JN115037.1,

JN407493.1, JN407492.1, JN407491.1, FJ917003.1, FJ980402.1, KF986154.1, KF986150.1, KC573848.1, KC573847.1, KC573846.1, KC573845.1, KC573844.1, KC573843.1, KC573842.1, KC573841.1, KC573840.1, GQ434714.1, GQ434713.1, KJ606359.1, KJ606358.1, KJ606356.1, KJ606354.1, KJ606352.1, KJ606350.1, AB604278.1, AB604277.1, AB604276.1, AB604248.1, AB604249.1, AB604289.1, AB604288.1, AB604287.1, AB604286.1, AB604281.1, AB604274.1, AB604273.1, AB604269.1, AB604265.1, AB604263.1, AB604262.1, AB604261.1, AB604260.1, AB604258.1, AB604247.1, AB604245.1, AB604244.1, AB604243.1, AB604242.1, AB604291.1, AB604290.1, AB604285.1, AB604284.1, AB604283.1, AB604282.1, AB604280.1, AB604279.1, AB604275.1, AB604272.1, AB604271.1, AB604270.1, AB604268.1, AB604267.1, AB604266.1, AB604264.1, AB604259.1, AB604257.1, AB604256.1, AB604255.1, AB604254.1, AB604253.1, AB604252.1, AB604251.1, AB604250.1, AB604246.1, AB604241.1, AB604240.1, AB604239.1, AB604238.1, AB604237.1, AB604236.1, AB604235.1, AB604234.1,

AB604233.1, AB604232.1, AB604231.1, AB604230.1, AB604229.1, AB604228.1, AB604227.1, AB604226.1, AB604225.1, AM042006.1, AM042005.1, AM042004.1, AM042003.1, AM042002.1, AM042001.1, AM042000.1, AM041999.1, AM041998.1, AM041997.1, AJ554222.1, AJ554227.1, AJ554226.1, AJ554225.1, AJ554224.1, AJ554223.1, AJ554221.1, AJ554220.1, AJ554219.1 and AJ554218.1.

43 of the above were used to construct phylogenetic tree of Morus including KF784875, KF784876, KF784877, KF784879, KF784881, KF784882, KF784883, KF784884, KF784885, KF784886, KF784887, KF784888, KF784889, KF784890, KF784891, KF784892, KF784893, KF784894, KF784895, KF784896, KF784897, HM623778, EU091563, HM747167, AM042004, HM747168, HM747170, HM747173, AY345145, AM041997, HQ144170, HQ144175, HQ144180, FJ980402, HM747171, HM747176, HM747169, AY345154, AM042006, AM042005, AM041999, AB564722, KF850474.
